# Moonlighting and physician residents’ compensation: is it all about money? A cross-sectional Brazilian study

**DOI:** 10.1590/1516-3180.2022.0187.R2.23082022

**Published:** 2022-10-24

**Authors:** Mário Luciano de Mélo Silva-Júnior, Pedro Augusto Sampaio Rocha-Filho

**Affiliations:** I MD, PhD. Professor, Medical School, Division of Neuropsychiatry, Universidade Federal de Pernambuco (UFPE), Recife (PE), Brazil; Professor, Neurology Unit, Medical School, Uninassau, Recife (PE), Brazil; Physician, Neurology Unit, Hospital da Restauração, Recife (PE), Brazil.; Uninassau, Medical School, Neurology Unit, Recife, PE, Brazil; Hospital da Restauração, Neurology Unit, Recife, PE, Brazil; II MD, PhD. Professor, Medical School, Division of Neuropsychiatry, Universidade Federal de Pernambuco (UFPE), Recife (PE), Brazil. Professor, Postgraduate Program in Neuropsychiatry and Behavioral Sciences (Posneuro), Universidade Federal do Pernambuco (UFPE), Recife (PE), Brazil; and Physician, Headache Clinic, Hospital Universitario Oswaldo Cruz, Universidade do Pernambuco (UPE), Recife (PE), Brazil.; Universidade Federal de Pernambuco, Recife, PE, Brazil; Universidade do Pernambuco, Hospital Universitario Oswaldo Cruz, Headache Clinic, Recife, PE, Brazil

**Keywords:** Workload, Internship and residency, Remuneration, Moonlighting, Duty hours, Medical residency, Effort-reward imbalance

## Abstract

**BACKGROUND:**

Moonlighting is a largely discussed, however under-explored, subject among physician residents.

**OBJECTIVES:**

To analyze the frequency of moonlighting and its related factors.

**DESIGN AND SETTING:**

This cross-sectional study enrolled medical residents from all geographical regions of Brazil.

**METHODS:**

A web-based structured closed-ended survey was applied that explored the frequency and type of moonlighting, residency programs characteristics, and psychological distress. The questionnaire was published on social networks.

**RESULTS:**

The completion rate was 71.4% (n = 1,419) and 37.7% were males aged 28.8 ± 3.2 (mean ± standard deviation) years, and 571 (40.2%) were post-graduate year (PGY) 1. There were residents from 50 medical specialties (the most common training area was clinical, 51.9%). A total of 80.6% practiced moonlighting, with an average weekly workload of 14.1 ± 9.4 h, usually overnight or in weekend shifts. Factors related to it were being PGY-2 or higher (adjusted odds ratio = 3.90 [95% confidence interval = 2.93–5.18], logistic regression), lower weekly residency duty hours (0.98 [0.97–0.99]), and a higher salary (1.23 [1.08–1.40]). In contrast, perception of a “fair/adequate” compensation was influenced by age (1.02 [1.01–1.02]), not being single (1.05 [1.01–1.10]), and residency duty hours (1.51 [1.22–1.88]). Depression, anxiety, diurnal somnolence scores, and work-personal life conflicts were not correlated with moonlighting status.

**CONCLUSION:**

Moonlighting frequency is high, and it is related to higher PGY, briefer residency duty hours, and the perception that remuneration should be higher. This study provides insights into the motivations for moonlighting and effort-reward imbalance.

## INTRODUCTION

In the case of physician residents, moonlighting refers to medical practice unrelated to training requisites. Residents have perceived positive effects of moonlighting, such as gain of autonomy, experience, and competence;^1^ however, the main motivation to do so seems to be financial.^2,3^

In Brazil, medical residency programs pay a remuneration of approximately R$ 36,000 per year (Brazilian currency, equivalent to 8,490 US dollars, considering the exchange during the period of our data collection, in 2019), and the residents should work 60 hours per week. This compensation is lower than that practiced in other South American countries, such as Colombia and Peru.^4,5^ For comparison purposes, the mean salary of an attending physician in Brazil is estimated to be R$ 229,500 (54,127 US dollars) per year, with a mean workload of 55 hours per week.^6^ Residents’ low remuneration, associated with living costs in large cities, the need to support family members, and debts from medical college, leads to a high proportion of residents seeking moonlighting.^7,8^ Studies addressing the relation between resident compensation, financial strain, and moonlighting practice are lacking.

In Brazil, as there are no standards on residents’ moonlighting, they can moonlight at any time in residency; however, the moonlighting hours do not count toward the requirement of 60 weekly hours in the residency program. In the United States (US), moonlighting hours must be included in the weekly limit of 80 h, and some programs do not allow or have specific standards for moonlighting.^2,3^

Research has been conducted on residency program duty hours and their negative impacts on residents’ health.^9,10^ However, data have related moonlighting to a better quality of life and satisfaction with work-life balance, as well as to reduced frequencies of stress and burnout.^3,11,12^ It is important to improve comprehension of these conflicting observations. Understanding the motivations for moonlighting practice, and its consequences on both residents’ learning and patient safety is warranted. Nonetheless, this is a poorly explored subject worldwide, and data on moonlighting and its related factors are scarce.

## OBJECTIVE

This study aimed to describe the frequency of moonlighting among a nationwide multi-specialty sample of physician residents in Brazil, as well as the related factors. Further, we aimed to analyze residents’ perceptions of the “fairness” of the current compensation they received.

## METHODS

This cross-sectional study enrolled a nationwide sample of medical residents from Brazil between November and December 2019. We developed and performed face validation, tested the questions for comprehension with a pilot of 20 residents, and assessed the ease-of-use of the final tool. We then conducted an online survey called, “How is your medical residency going?,” which aimed to assess general questions about residency training, using 46 questions over four pages. This was the first study to enroll residents from all regions of Brazil and was primarily exploratory. Details and primary analyses of this study have already been published.^13,14^

The questionnaire was outreached on social networks (Facebook and Instagram, in pages/profiles of medical residents’ associations). To ensure that only medical residents answered the survey, we had an obligatory button, “I confirm that I am a medical resident currently,” displayed before the questionnaire.

The STROBE reporting guidelines were followed in this study. The Ethics Committee of Universidade Federal de Pernambuco (UFPE) approved this study before data collection (Approval number: 3.314.833 on May 9, 2019). All individuals provided consent, and no benefits were offered or given to participate.

### Moonlighting

Moonlighting was defined as performing any paid medical activity unconnected to residency program requirements. We examined the frequency and type (oncalls or outpatient care) of this activity. According to Brazilian laws, medical residents are allowed to moonlight at any time during residency training; moonlighting hours are not included in the residency program duty hours.

### Residency salary

We enquired regarding the residency salary (monthly financial value received by the residents from the institutions that provide the residency program) on two topics. Residents’ judgment on the amount received (Is the current value of the residency salary fair/adequate?); and the residents’ judgment on how much amount would be appropriate (what would be the fair/adequate value of the residency salary?).

In case of individuals who practiced moonlighting, we also asked about the impact of a hypothetical scenario in which they received the amount believed to be “fair/adequate” (if you received the amount mentioned in the previous question, what would you do about moonlighting?). All questions were closed-ended.

### Psychological distress

Validated tools were used to measure anxiety, depression, and diurnal somnolence. Patient Health Questionnaire-4 (PHQ-4) is a screening method using four Likert-type questions (two for depression and two for anxiety), with scores ranging from 0 to 3 (higher scores indicate a higher chance of these conditions). Individuals who scored ≥ 3 had a positive screening result for a specific condition.

Day-time sleepiness was assessed using the six-item Brazilian version of Epworth Sleepiness Scale, each Likert-type question score ranged from 0 to 3 (higher scores indicated higher diurnal somnolence). Individuals who scored ≥ 10 had positive screening results for diurnal somnolence.

Work-personal life conflicts were assessed by the affirmation “My routine in this medical residency program allows me enough time for my personal and family activities.” It was a five-item Likert-type response ranging from “strongly disagree” to “strongly agree.” Individuals who answered “strongly disagree” or “disagree” were classified as having work-personal life conflicts.

### Residency program and socio-demographic aspects

We included questions on residency program characteristics (duty hours, training area [clinical, surgical, or diagnostic], post-graduate year [PGY], and geographic region of training). Personal data included age, sex, marital and child status, and weekly leisure hours (time spent with himself/herself [hobbies, physical exercises, beauty care, etc.]). We questioned whom the residents lived with, and if they had to move to participate in the residency program.

### Statistical analysis

According to the responses to moonlighting questions, individuals were categorized into the moonlighting (any type or workload) or control (no reported moonlighting at all) groups.

Discrete variables are expressed as mean and standard deviation, and comparisons between the two groups were performed with Mann–Whitney or student’s t-test, according to parametric distribution (Kolmogorov–Smirnov test). For comparisons between more than two groups, we applied the Kruskal–Wallis test with Dunn’s correction. Qualitative variables are expressed as percentages, and contingency analyses were conducted using the Chi-square test. Correlations were expressed using Spearman’s rho.

To analyze independent (dichotomized or discrete) variables affecting moonlighting, we applied binary logistic multiple regression. Poisson regression model was used to assess factors influencing the compensation that residents stated they should receive. All variables with P < 0.20 in bivariate analysis were included in the model, and a backward stepwise process was performed (excluding the factors with higher P-values on Wald test) until all factors were at P < 0.05.

Once all answers were obtained to go ahead with the survey (except those that might identify the volunteers), our missing data were low (< 0.1%). Individuals with missing data were excluded from specific analyses. For sensitivity analyses, we identified multivariate outliers using the Mahalanobis test, excluded those individuals (n = 43), and reanalyzed the data.

All analyses were performed using SPSS (Armonk, New York, United States) v25 for MacOS. A P value of 0.05 was considered statistically significant for all analyses. We did not calculate the sample size before data collection.

## RESULTS

Our link received 1,989 clicks, of which 1,421 individuals completed the survey (71.4% completion rate). Two participants were excluded because of conflicting answers (n = 1,419).

### Study population

Our sample was composed of 535/1,419 (37.7%) males, with a mean age of 28.8 ± 3.2 years. Majority had no children (1,292/1,419, 91.1%), were single (978/1,419, 68.9%), and needed to move to participate in residency (913/1,419, 64.3%). Regarding residency-related aspects, 40.2% (571/1,419) were PGY-1, 29.7% were PGY-2, and 30.1% were PGY-3 or higher. The clinical training area was the most common (736/1,419, 51.9%), followed by surgical (43.0%), and diagnostic (5.1%).

### Moonlighting

Majority (1,140/1,419, 80.3%) of the residents practiced moonlighting, with an average weekly workload in these activities of 14.1 ± 9.4 h. Table 1 compares the socio-demographic data according to moonlighting status.

**Table 1:** Bi- and multivariate analysis of demographic, psychological distress, and program-related characteristics, according to moonlighting status

Variables*	Moonlighting (n = 1,140)	Controls (n = 279)	P	aOR (95% CI)	P
Moonlighting**	14.1 ± 9.4	–	–	–	–
Age	29.0 ± 3.2	28.0 ± 2.9	< 0.001	–	–
Male sex	443 (39.4)	92 (33.2)	0.062	–	–
Have child/children	111 (9.7)	16 (5.7)	0.035	–	–
Single	765 (67.1)	213 (76.3)	0.003	–	–
Moved to take this residency training	724 (63.5)	189 (67.7)	0.209	–	–
Live alone	431 (37.8)	109 (39.1)	0.731	–	–
Geographic area, South	773 (67.8)	199 (71.3)	0.281	–	–
Post-graduation year 2 or higher	757 (66.4)	88 (31.5)	< 0.001	3.90 (2.93–5.18)	< 0.001
Residency duty**	68.8 ± 14.9	75.0 ± 18.8	< 0.001	.98 (0.97–0.98)	< 0.001
Clinical training area	613 (53.8)	123 (44.1)	0.004	–	–
Leisure time**	7.4 ± 6.5	6.4 ± 6.6	0.002	–	–
Work-personal life conflicts	891 (78.2)	212 (76.0)	0.424	–	–
Epworth sleepiness positive screen	715 (62.7)	181 (64.9)	0.534	–	–
PHQ-4 positive screen	564 (49.5)	135 (48.4)	0.789	–	–
“Fair” value of salary#	6.9 ± 1.9	6.5 ± 1.9	0.002	1.23 (1.08–1.40)	0.002

OR = odds ratio; CI = confidence interval; PHQ-4 = Patient Health Questionnaire-4. aOR = adjusted odds ratio.

* mean±standard deviation, or n (%).

** Hours per week

# value in thousand reais (Brazilian currency). aOR refers to logistic regression model. Sensitivity analysis did not significantly change the results.

Variables independently related to moonlighting were being PGY-2 or higher, lower weekly residency duty hours (a mean difference of 6.2 h, P < 0.001), and considering higher values of salary as “fair/adequate.” Moreover, individuals who did moonlight were older, non-single, male, and parents, and had a slightly longer leisure time (mean difference of 1.0 h, P = 0.002) than those who did not, although these did not persist after adjustment for confounders.

Epworth sleepiness, PHQ-4 scores, and frequency of work-personal life conflicts did not differ between the groups. It is worth mentioning that the frequency of positive screening was high.

The residents generally moonlighted overnight and/or in weekend shifts (1,100/1,140, 96.5%), but almost one-quarter (267/1,140, 23.4%) practiced outpatient care. Table 2 depicts these data and the residents’ judgments regarding salary values.

**Table 2:** Frequency of moonlighting, perception of the value of the medical residency salary, and the impact of a possible adjustment of the salary value on moonlighting

Variable		Result (n, %)
**Type of moonlighting** *		
	Overnight and/or weekend shifts	1,100 (77.5)
	Outpatient care	267 (18.8)
	No moonlighting	279 (19.7)
**Is the current value of the residency salary fair/adequate?** *		
	No, because it does not match with my quantity of working hours	1194 (84.1)
	No, because it is not proportional to the complexity of the tasks/activities that I perform	959 (67.6)
	No, because it is not enough to support myself	898 (63.3)
	No, because it is not equivalent to the income of other governmental programs, such as “MaisMédicos”**	749 (52.8)
	No, because it is not equivalent to the income of the other medical staff	308 (21.7)
	Yes, because the hospital has additional costs to having residents	10 (0.7)
	Yes, because it is enough to support myself	7 (0.5)
**What would be the fair/adequate value of the residency salary?**		
	The current value is fair	7 (0.5)
	1/3 more (about 4 thousand reais)	67 (4.7)
	2/3 more (about 5 thousand reais)	362 (25.5)
	Double (about 6 thousand reais)	482 (34.0)
	Triple (about 9 thousand reais)	344 (24.2)
	More than the triple	157 (11.1)
**If you received the amount mentioned in the previous question, what would you do about moonlighting?** †		
	I would not work in any moonlighting, at all	783 (68.7)
	I would decrease my quantity of moonlighting	340 (29.8)
	I would not change my routine of moonlighting	17 (1.5)

* The sums are greater than 1419, because each individual could check more than one option.

** A Brazilian governmental program intended to increase the number of physicians around Brazilian territory.

† Individuals who do moonlighting (n = 1.140).

### Residency salary perception

Most residents (1,412/1,418, 99.5%) believed that the compensation they received was not “fair/adequate”. The main reasons for this are described in Table 2, which included “high workload” (84.1%), “complexity of the tasks performed” (67.6%), and “insufficient value to support him/herself” (63.3%).

The mean “fair” value was considered to be R$ 6.8 ± 2.0 thousand per month (equivalent to US$ 19,245 per year, considering the exchange at the time of data collection).

**Figure 1:**
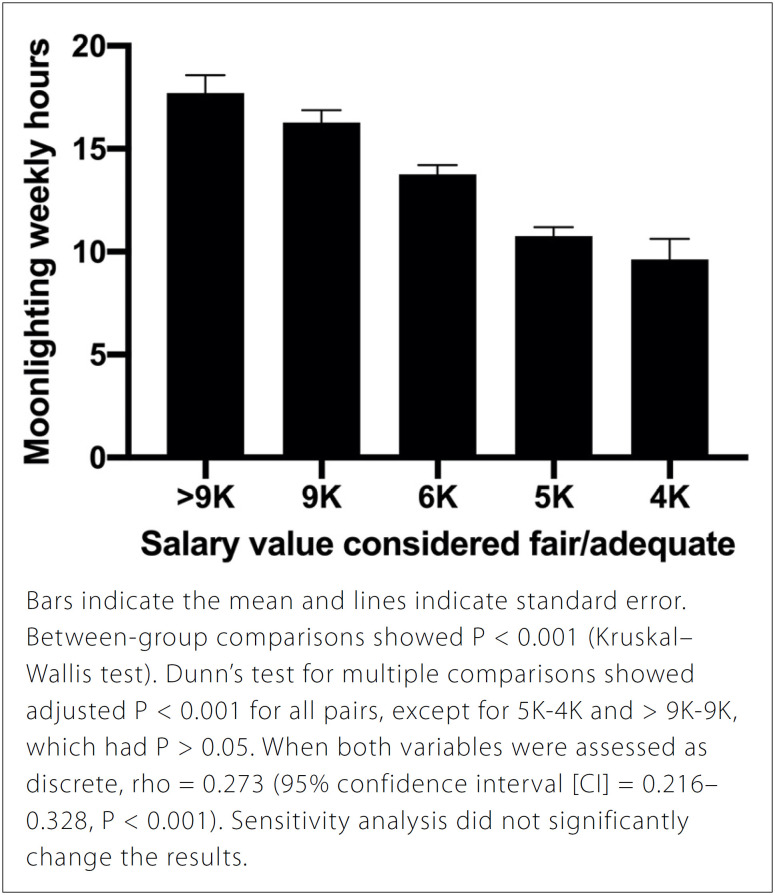
Compensation value considered fair/adequate by the residents in relation to mean moonlighting hours per week (n = 1.140).

We found positive correlations between how much would be the “fair/adequate” salary and the weekly duty hours spent practicing moonlighting (rho = 0.273, 95% confidence interval [CI] = 0.216–0.328, P < 0.001, Figure 1), and the number of motives to justify the “unfairness” of the current compensation (rho = 0.261, 95% CI = 0.210–0.310, P < 0.001).

Regarding the hypothetical scenario in which residents who practice moonlighting would receive the compensation cited as “fair/adequate,” majority (783/1,140, 68.7%) reported that they would stop moonlighting, and only 1.5% would not change their moonlighting routine in this situation.

In Poisson regression model, the compensation value reported as “fair/adequate” was influenced by higher age (1.02 [95% CI = 1.01–1.02], P < 0.001), longer residency duty hours (1.51 [1.22–1.88], P < 0.001), and not being single (1.05 [1.01–1.10], P = 0.024).

## DISCUSSION

This is the first study addressing moonlighting and related factors in Brazil, and the first in the world correlating moonlighting workload to salary perceptions. Our data show that more than 80% of residents moonlight, and the time spent in these activities is high (approximately 14 h per week). Moonlighting was related to higher PGY, briefer residency duty hours, and considering higher values of remuneration as “fair/adequate.” Almost all (99.5%) surveyed residents thought that the current Brazilian residency salary is not “fair/adequate,” mainly because of the high workload and complexity of the tasks performed. Moonlighting was not associated with psychological distress. These data shed some light on understanding of effort-reward imbalance in residents, moonlighting practices, and related factors.

In line with our data, other studies^15,16^ have shown that a higher PGY increases the odds of moonlighting. We hypothesized that the confidence and skills obtained during residency training, in tandem with the professional relationships built in this process, are central factors in opting for moonlighting. Moreover, working hours of PGY-1 are usually longer,^16^ which hampers this possibility. However, we did not find significant differences in moonlighting practice and specific areas of training (clinical versus surgical or diagnostic areas), which is different from others.^11^

The moonlighters expected higher compensation values. We found an association between expected compensation and moonlighting workload, and a significant proportion (98.5%) of residents stated that in a hypothetical scenario of receiving a “fair/adequate” residency program salary, they would stop or reduce duty hours in moonlighting. In addition, moonlighters have a higher chance of having children and being married. The hypothesis that perceived financial strain (present or future, presumed) is the main cause of moonlighting, appears to fit our model. Indeed, studies have shown that moonlighting increases income,^2,17^ and a large section of literature agrees with that.^1,14,18–20^ In contrast, it is worth noting that there are other motivations for moonlighting, such as maximizing learning, getting autonomy and experience, and improving procedural skills.^1,11,15,19^

The frequency of moonlighting depends on other factors beyond those mentioned above, such as specialty and hospital demands^21^ and workload of residency program training.^16^ The mean moonlighting duty hours per week found by us (mean 14.1 h) were far higher than that in the US literature (average 4 to 8 h,^1,3^ although one study pointed to 20.2 h in a small sample of surgical residents),^19^ probably owing to the longer (80 h compared to 60 h in Brazil) duty hours requirement in the US.

Individuals who did not practice moonlighting had higher workload in the residency program (however, both groups had mean duty hours higher than Brazilian standards) and less leisure time. High-intensity training programs may hinder residents from engaging in moonlighting and leisure activities, but we did not assess this program’s aspect. We believe that PHQ-4 scores and the frequency of diurnal somnolence and work-personal life conflicts were similar between the groups because of the counterbalancing effect of those factors. Some studies have found that moonlighters have a better quality of life and work-life balance,^12^ as well as smaller frequencies of burnout^17^and stress,^11^ although others did not.^3,14^ Poor sleeping patterns due to moonlighting were not observed, although some data^2^ pointed it as the main issue of moonlighting.

Perhaps individuals more prone to moonlighting consider the presumed training workload when opting to join a specific specialty or hospital.^11^ Another possibility is that individuals who cope better with residency demands have a higher chance of engaging in moonlighting; these hypotheses are not mutually exclusive. However, these interactions should be cautiously interpreted. Moreover, the impact of moonlighting on patient safety, frequency of medical errors, and residents’ learning is not fully understood. We found studies that did not find differences in objective^22^ or subjective^23^ evaluations of learning regarding moonlighting status.

It should be mentioned that the perception of the “fair/adequate’ value of salary depended on higher age, not being single, and longer residency duty hours.

Main reasons for considering the current value “inadequate/unfair” were, indeed, the quantity (reflected on duty hours) and complexity of the tasks performed, configuring an effort-reward imbalance setting. It is worth noting that the cited workload of moonlighting pays, on average, what most residents said it would be fair to receive (i.e., an additional 66% to 100% of the current compensation);^24^ and that higher age and not being single may be related to the need for a higher income – this aspect has already been described^11,16^ and may be a motivation for moonlighting.

Our study had several limitations. It was outreached in pages of the Brazilian Association of Residents on social networks; hence, it was a convenience sample, and we might have a selection bias. However, our sample is similar to census data^25^ regarding age, sex, area of training, and geographic distribution. According to this census^25^, the total number of medical residents in 2018 was 35,187; we achieved 4.0% of this population. Nevertheless, it is worth mentioning that this sample could not be reached by our approach. We could not control the number of responses provided by a specific participant in this survey. All variables were self-reported. We did not ask about the debt burden; hence, we were not able to assess its relationship with moonlighting.

## CONCLUSION

Moonlighting frequency is high and is related to higher PGY, briefer residency duty hours, and the perception that the salary should be higher. Most residents think that they earn compensation lower than deserved, based on the high workload and complexity of the tasks performed. The “fair/adequate” value of the salary was associated with higher age, not being single, and longer residency duty hours.

This study provides insights into the motivations for moonlighting and effort-reward imbalance among residents. The impact of moonlighting on the learning of residents and patient safety should be addressed in further studies.
